# Phosphoinositide-3 Kinase Inhibition Modulates Responses to Rhinovirus by Mechanisms that Are Predominantly Independent of Autophagy

**DOI:** 10.1371/journal.pone.0116055

**Published:** 2014-12-26

**Authors:** Saila Ismail, Clare A. Stokes, Elizabeth C. Prestwich, Rebecca L. Roberts, Jatinder K. Juss, Ian Sabroe, Lisa C. Parker

**Affiliations:** 1 Academic Unit of Respiratory Medicine, Department of Infection and Immunity, Faculty of Medicine, Dentistry and Health, University of Sheffield, Beech Hill Road, Sheffield, S10 2RX, United Kingdom; 2 Department of Medicine, University of Cambridge School of Clinical Medicine, Addenbrooke's and Papworth Hospitals, Cambridge, CB2 2QQ, United Kingdom; School of Medicine, University of Belgrade, Serbia

## Abstract

Human rhinoviruses (HRV) are a major cause of exacerbations of airways disease. Aspects of cell signalling responses to HRV infection remain unclear, particularly with regard to signalling via PI3K, and the PI3K-dependent pathway, autophagy. We investigated the roles of PI3K and autophagy in the responses of epithelial cells to major and minor group HRV infection. The PI3K inhibitor 3-MA, commonly used to inhibit autophagy, markedly reduced HRV-induced cytokine induction. Further investigation of potential targets of 3-MA and comparison of results using this inhibitor to a panel of general and class I-selective PI3K inhibitors showed that several PI3Ks cooperatively regulate responses to HRV. Targeting by siRNA of the autophagy proteins Beclin-1, Atg7, LC3, alone or in combination, or targeting of the autophagy-specific class III PI3K had at most only modest effects on HRV-induced cell signalling as judged by induction of proinflammatory cytokine production. Our data indicate that PI3K and mTOR are involved in induction of proinflammatory cytokines after HRV infection, and that autophagy has little role in the cytokine response to HRV or control of HRV replication.

## Introduction

Rhinoviruses are a leading cause of exacerbations of asthma and chronic obstructive pulmonary disease (COPD) [1]. The initial responses to human rhinovirus (HRV) are mediated by the endosomal pattern recognition receptor, TLR3, followed by additional signals from the cytoplasmic pattern recognition receptors, retinoic acid inducible gene-1 (RIG-I) and melanoma differentiation associated protein 5 (MDA5) [2]. Further layers of response coordination are provided by activation of phosphoinositide-3 kinase (PI3K) signalling [3]–[6], though the PI3K classes involved in regulation of HRV signalling are not known.

TLR3 recognises double-stranded viral RNA (dsRNA), generated during HRV replication. The early signalling pathways involved in responses to HRV, and the mechanism by which dsRNA reaches the endosome, remain incompletely understood. Autophagy is a PI3K-dependent pathway that involves the sequestration of cytoplasmic material and organelles in autophagosomes, followed by their disassembly and destruction through the endosomal/lysosomal pathway [7]. Autophagy participates in the control of various viral infections (reviewed in [7]). In dendritic cells, autophagy delivers viral replication products from the cytoplasm to TLR7-containing endosomes [8]. However, autophagy has not yet been shown to be a major mechanism delivering double-stranded RNA intermediates to TLR3-containing endosomes. Furthermore, the roles of autophagy in HRV infection remain controversial. In one study, HRV-2 infection was not associated with induction of autophagy [9]. In contrast, HRV infection has been associated with autophagosome formation [10] and recent work has suggested that autophagy is necessary for maximal viral replication of HRV-2 and HRV-14 [11].

Dissecting the roles of PI3K and autophagy in responses to HRV infection is additionally complicated by the recent finding that the main class III PI3K inhibitor traditionally used to selectively target the autophagic pathway, 3-methyladenine (3-MA), has been shown to inhibit other pathways such as class I PI3K [12], [13].

We therefore set out to investigate the extent to which responses to HRV were dependent upon autophagy and PI3K signalling. We found that knockdown of autophagy proteins had little or no impact on the induction of proinflammatory cytokines by HRV infection or significant consequences for rhinoviral replication, although we note that low levels of autophagy proteins may permit some responses to still function. We also determined that multiple PI3K isoforms contributed to responses to HRV infection, and we suggest a role of mTOR in the regulation of responses to HRV.

## Methods

### Epithelial cells

We studied the immortalised human bronchial epithelial cell line BEAS-2B. These cells retain characteristics of normal airways epithelial cells [14], [15]. Cells were from the American Type Culture Collection (ATCC), maintained in RPMI 1640 containing 2 mM L-glutamine, 10% fetal calf serum (FCS) and antibiotics (cell culture reagents from Invitrogen, FCS [endotoxin levels of 0.5 EU/ml] from Promocell) (complete media).

### HRV stocks

HRV minor group serotype 1B (RV-1B) and major group serotype 16 (RV-16) were propagated in HeLa Ohio cells (from the European Collection of Cell Culture), yielding stocks containing on average 2×10^7^ 50% tissue culture infective doses (TCID_50_)/ml and 3×10^7^ TCID_50_/ml of RV-1B and RV-16, respectively [16], [17], determined by viral cytopathic effect (CPE) assay. Neutralisation using serotype-specific antibody (Ab) (ATCC) was carried out to confirm viral identities.

### Infection and stimulation of epithelial cells

BEAS-2B cells were grown to ∼95% confluence in 12 well plates, and then cultured overnight in RPMI 1640/2% FCS and antibiotics (infection media) prior to infection. Cells were infected with HRV at the indicated TCID_50_/ml for 1 h at room temperature with gentle shaking. Virus was then removed, cells were washed twice with media, and 1 ml of infection media was added/well. Cells were cultured at 37°C for 6 h or 24 h, after which supernatants or cell lysates were harvested.

To examine responses to agonists, confluent epithelial cells were stimulated with polyinosinic:polycytidylic acid [poly(I:C), a synthetic mimic of dsRNA, from Invivogen], IL-1β, or interferon-β (IFNβ) (PeproTech EC). After 24 h, supernatants or cell lysates were harvested.

### Small molecule inhibitors

Studied PI3K inhibitors were from Cayman Chemical unless noted, and comprised 3-MA (Sigma-Aldrich), LY294002 (Calbiochem), PIK-75, TGX-221, IC87114, AS605240, or PI-103. Rapamycin (an mTOR inhibitor) used was from Tocris Bioscience. Inhibitors were added at the time of stimuli (for cytokines, poly(I:C)), or immediately after viral infection.

### siRNA

BEAS-2B cells were grown to ∼70% confluence in 12-well plates. Beclin-1 (Bec1), Atg7, light chain 3 (LC3) and Vps34 were knocked down by Dharmacon siRNAs (Thermo Scientific) transfected with Lipofectamine2000 (Invitrogen) as described [16]. ON-TARGET*plus* SMARTpool siRNAs were used for *Bec1* (L-010552-00), *Atg7* (L-020112-00), *Vps34* (L-005250-00), *p85α* (L-003020-00) and *p85β* (L-oo3021-00); and siGENOME SMARTpool siRNAs for *LC3A* (M-013579-00) and *LC3B* (M-012846-01), the latter two combined to knockdown both LC3A and LC3B simultaneously. All siRNAs were used at 100 nM. Appropriate scrambled (scr) controls were selected. Cells were incubated for 48 h before being infected with HRV or stimulated with agonists.

In one series of n = 4 experiments, parallel cell counts were performed to facilitate interpretation of TCID_50_ values for added virus. In this set of experiments, an average of 169,000 cells were present pre-siRNA (transfection at 70–75% confluence). Immediately before addition of HRV, wells showed ≥95% confluence by visual inspection, and contained an average of 657,000 cells/well (media-treated), 520,000 cells/well (scrambled siRNA treated) and 517,000 cells/well (transfected with siRNA to Beclin-1).

### ELISA

CXCL8 and CCL5 proteins in cell-free supernatants were quantified by ELISA, using matched Ab pairs (R&D systems) at optimised concentrations [18]. Detection limits for CXCL8 and CCL5 were 78 and 312.5 pg/ml, respectively.

### Quantification of cell death

The amount of lactate dehydrogenase (LDH) released by the dead cells was measured using CytoTox 96 Non-Radioactive Cytotoxicity Assay (Promega).

### PCR

RNA was extracted using TRI Reagent (Sigma) with DNase I digestion of contaminating DNA (Ambion). cDNA was synthesised using a reverse transcriptase kit (Applied Biosystems). Primer-probe sets for quantitative PCR (qPCR) were from Sigma-Aldrich (HRV and IFNβ) or Applied Biosystems for GAPDH [16], [17]. PCR master mix was from Eurogentec (Southampton, United Kingdom). Samples were quantified against serial dilutions of standards of plasmids containing the target sequences to provide copy number.

### Western blot

Lysates and western blotting was performed as described [16], [17]. Abs were from Abcam for Bec1, p85α and p85β; Cell Signaling Technology for Atg7, LC3, Vps34, phospho-p70S6K (Thre 399), phospho-p70S6K (Thre 421/Ser424) and total p70S6K; and Sigma-Aldrich for actin. Detection was by a horseradish peroxidase coupled secondary Ab (Cell Signaling Technology). Images were acquired using a Biorad Chemidoc XRS+ system. Densitometric analysis was conducted using NIH ImageJ software.

### Immunohistochemistry

Epithelial cells were grown on glass cover slips prior to transfection. After knockdown of indicated genes, cells were starved for 2 hours in Hank's Balanced Salt Solution (HBSS; Sigma-Aldrich). Cells were fixed with ice-cold 100% methanol, and stained for endogenous LC3 using anti-LC3B (Sigma-Aldrich). Secondary antibodies used were Alexafluor 488 (Molecular Probes). Nuclei were stained with 1× PBS containing 0.5 µg/ml 4′,6 diamidino-2-phenylindole (DAPI; Sigma-Aldrich) and mounted on slides with Fluoromount (Southern Biotech). Staining was visualised by an Olympus BX61 fluorescence microscope.

### Statistical analysis

Data are presented as mean ± SEM of at least three independent experiments on separate BEAS-2B cell culture passages and were analysed using ANOVA with the indicated post-test using Prism v6.0 (GraphPad Inc).

## Results

### 3-methyladenine inhibits cytokine production induced by HRV infection

**Figure 1:**
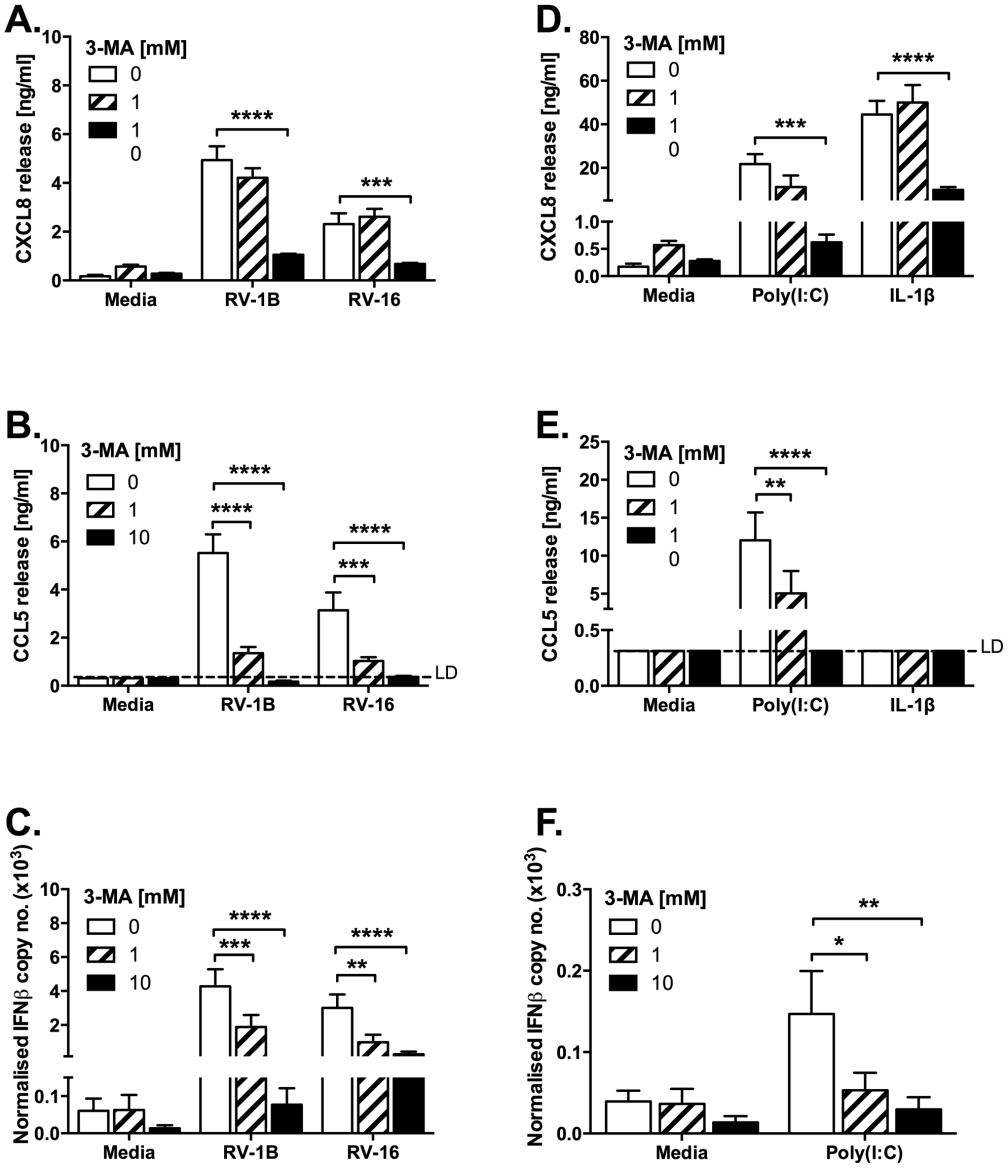
3-MA inhibits proinflammatory cytokine production induced by HRV infection. BEAS-2B cells were infected with RV-1B or RV-16 at 1×10^7^ TCID_50_/ml for 1 h (following which supernatants were replaced with media) (A, B, C); or stimulated with poly(I:C) (1 µg/ml) or IL-1β (1 ng/ml) (D, E, F). Cells were then immediately treated with 3-MA at the indicated concentrations and cultured for 24 h. Cell-free supernatants were collected, and CXCL8 (A, D) and CCL5 (B, E) release was measured by ELISA. RNA was extracted and reverse transcribed for qPCR analysis of IFNβ mRNA expression (C, F), with IFNβ copy number normalized to the GAPDH copy number as a loading control. Data shown are mean ± SEM of *n* = 5, where each replicate was performed on a separate passage of cells. Significant differences are indicated by *, *p*<0.05; **, *p*<0.01; ***, *p*<0.001 and ****, *p*<0.0001, analysed by two-way ANOVA with Bonferroni's post-test. Media controls shown on the left and right panel sets are the same. LD  =  limit of detection.

**Figure 2:**
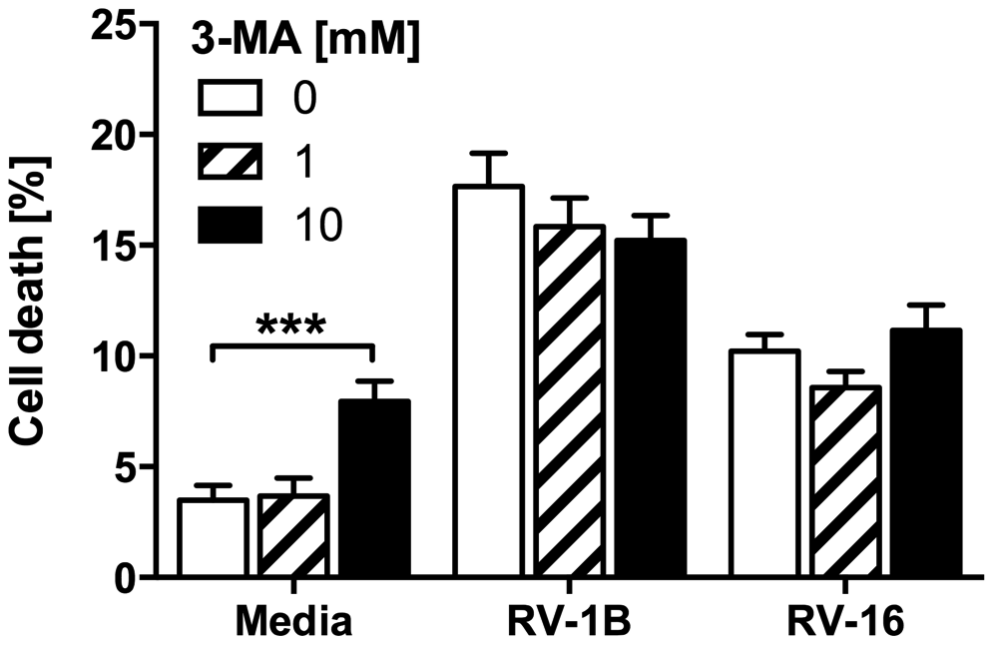
3-MA treatment does not increase the cell death of HRV-infected cells. BEAS-2B cells were infected with RV-1B or RV-16 at 1×10^7^ TCID_50_/ml for 1 h (following which supernatants were replaced with media). Cells were then immediately treated with 3-MA at the indicated concentrations and cultured for 24 h. Percentage of cell death was measured by LDH assay. Data shown are mean ± SEM of *n* = 4, where each replicate was performed on a separate passage of cells. The only significant difference on the chosen statistical testing is indicated by ***, *p*<0.001, analysed by two-way ANOVA with Bonferroni's post-test.

We first determined whether 3-MA, a class III PI3K inhibitor traditionally used as a selective inhibitor of the autophagy pathway, modulated cytokine responses in response to infection of BEAS-2B epithelial cells. To broaden relevance of our data we studied both minor and major group HRVs (RV-1B and RV-16 respectively), and compared these responses with the synthetic TLR agonist poly(I:C). In keeping with our previous studies [16]–[18], CXCL8 was chosen as it is generated upon activation of NF-κB and mitogen-activated protein kinase (MAPK)-dependent proinflammatory signaling, and as the principal mediator responsible for neutrophil recruitment is highly relevant to the pathology of asthma and COPD. CCL5 was selected as a typical IFN-stimulated gene (ISG) product generated downstream of IFN-α/β/λ production, indicative of activation of the IFN pathways. 3-MA treatment reduced production of CXCL8 and CCL5 protein and IFNβ mRNA from cells infected with RV-1B or RV-16 (Fig. 1). Reductions of CXCL8 protein were also mirrored by reductions in reduction in HRV-induced CXCL8 mRNA (data not shown), confirming that we were interrupting new transcription of inflammatory cytokines rather than release of preformed proteins stores. In our experiments, a TCID_50_ of 1 ×10^7^ corresponded to an MOI of approximately 3. The inhibitory actions of 3-MA were more marked on generation of IFNβ and CCL5 than CXCL8. We determined if 3-MA could also inhibit signalling in response to poly(I:C), a mimic of viral RNA that might also activate autophagy, and to IL-1β, which we did not expect to require activation of autophagy to signal. 3-MA inhibited CXCL8, CCL5, and IFNβ production induced by poly(I:C), in a dose-dependent pattern similar to its actions on HRV-induced inflammation. However, the higher tested concentration of 3-MA also inhibited the induction of CXCL8 production by IL-1β. These data suggested that 3-MA may exhibit off-target actions at the frequently exploited concentration of 10 mM. These effects were not the result of altered cell death, since 3-MA treatment did not measurably increase cell death beyond that caused by HRV infection alone (Fig. 2).

### Involvement of PI3Ks in responses to HRV

The class III PI3K inhibitor, 3-MA, inhibited CCL5 and IFN production induced by HRV and poly(I:C), and at high concentrations inhibited CXCL8 production induced by IL-1β (Fig. 1). In the light of recently published data [12], [13], we considered that its actions might be mediated by inhibition of more than one PI3K. We therefore compared 3-MA's actions with the actions of a broad PI3K inhibitor, LY294002, and a panel of selective class I PI3K inhibitors, chosen for well-characterised selectivity against specific PI3K isoforms.

Concentrations of selective class I inhibitors were chosen as being approximately 10-fold above the published IC_50_ values and preliminary experiments established these chosen concentrations were without noticeable cell toxicity (data not shown). We used the following inhibitors: LY294002, a general PI3K inhibitor [19]. PIK-75 at 0.1 µM, predominantly inhibiting class I p110α (IC_50_ 0.006 µM) and to a lesser degree p110γ (IC_50_ 0.08 µM) [20]. TGX-221 at 0.1 µM, predominantly inhibiting class I p110β (IC_50_ 0.007 µM) and to a lesser degree p110δ (IC_50_ 0.1 µM) [21]. IC87114 at 5 µM, inhibiting class I p110δ (IC_50_ 0.5 µM) [21]. AS605240 at 0.1 µM, predominantly inhibiting class I p110γ (IC_50_ 0.008 µM) and to a lesser degree p110α (IC_50_ 0.06 µM) [21]. PI-103 at 0.5 µM, a pan class I inhibitor [21].

**Figure 3:**
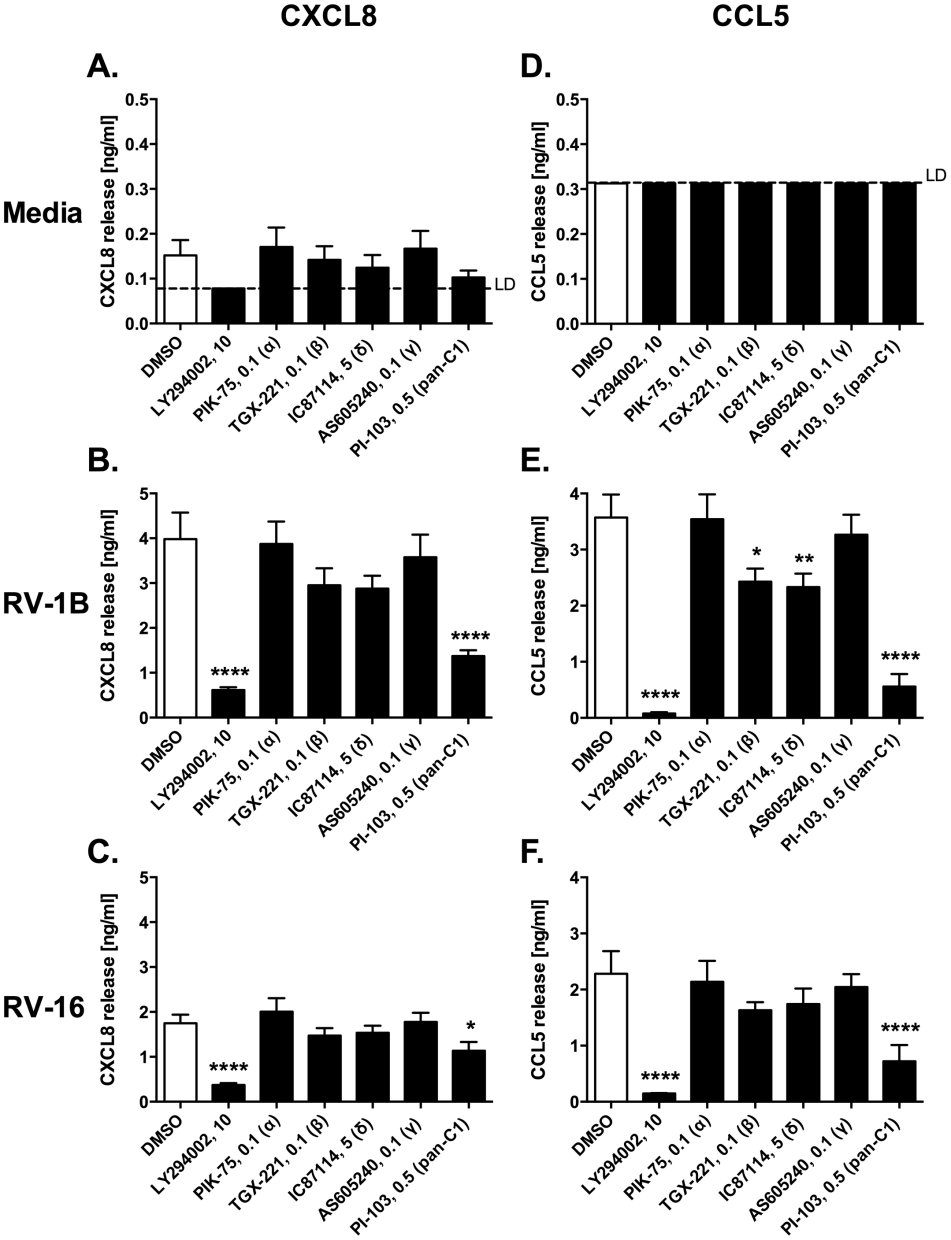
Effects of isoform-selective class I PI3K inhibitors on HRV-induced cytokine release. BEAS-2B cells were left uninfected (A, D); or infected with RV-1B (B, E) or RV-16 (C, F) at 1×10^7^ TCID_50_/ml for 1 h (following which supernatants were replaced with media). Cells were then immediately treated with DMSO control, or with the indicated PI3K inhibitors (the numbers shown after inhibitor names indicate the concentrations used in µM); and cultured for 24 h. Cell-free supernatants were collected, and CXCL8 (A, B, C) and CCL5 (D, E, F) release was measured by ELISA. Data shown are mean ± SEM of *n* = 4, where each replicate was performed on a separate passage of cells. Significant differences are indicated by *, *p*<0.05; **, *p*<0.01; and ****, *p*<0.0001 (versus DMSO control), analysed by one-way ANOVA with Tukey's post-test [Abbreviations: C1  =  class I inhibitor; LD  =  limit of detection].

**Figure 4:**
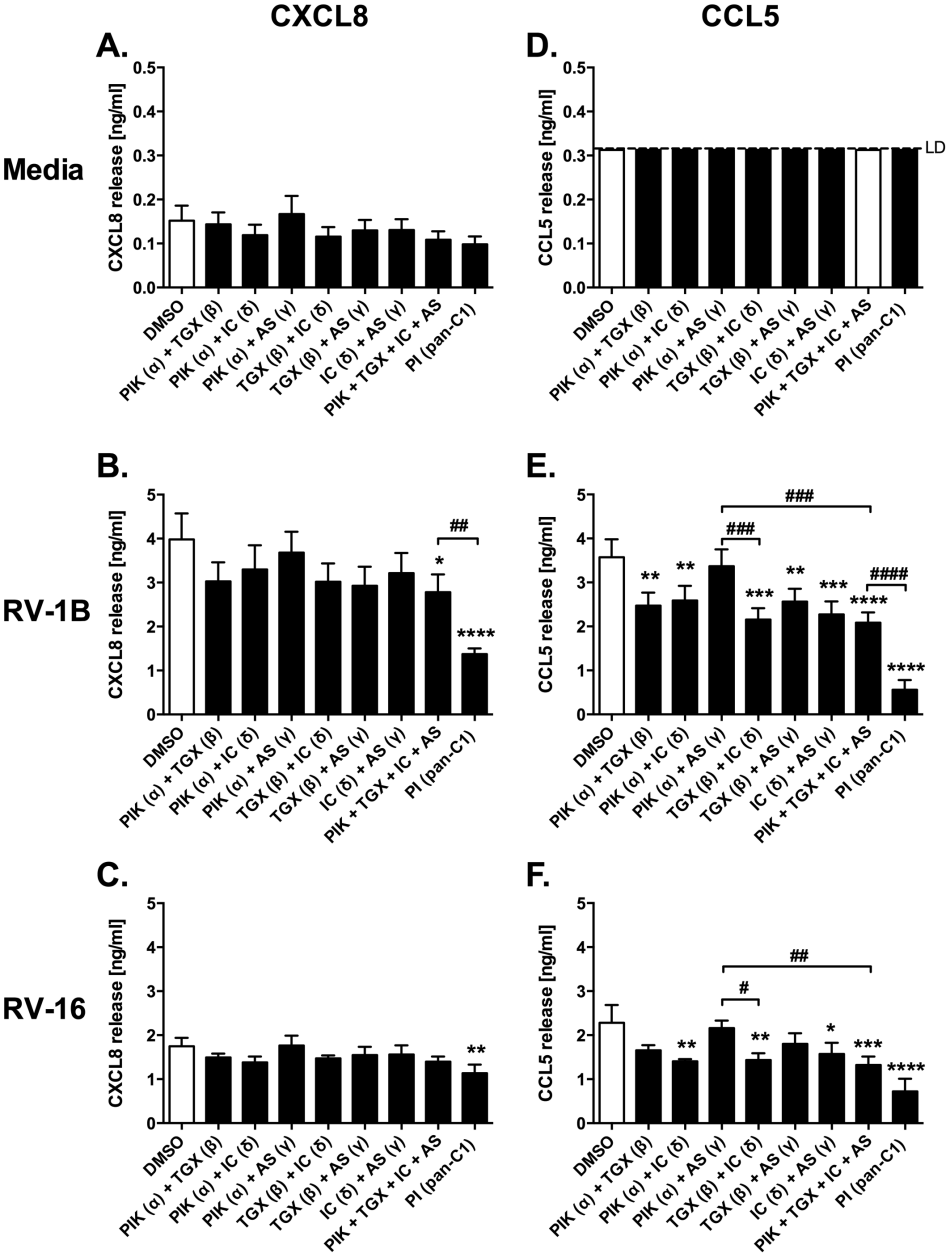
Effects of combinations of multiple isoform-selective class I PI3K inhibitors on HRV-induced cytokine release. BEAS-2B cells were left uninfected (A, D); or infected with RV-1B (B, E) or RV-16 (C, F) at 1×10^7^ TCID_50_/ml for 1 h (following which supernatants were replaced with media). Cells were then immediately treated with DMSO control, or with PIK-75 (PIK, 0.1 µM), TGX-221 (TGX, 0.1 µM), IC87114 (IC, 5 µM), AS605240 (AS, 0.1 µM) or PI-103 (PI, 0.5 µM), individually or in combination; and cells were then cultured for 24 h. Cell-free supernatants were collected, and CXCL8 (A, B, C) and CCL5 (D, E, F) release was measured by ELISA. Note that the data for DMSO controls and for the PI-103 alone are the same experimental data that are also displayed in Fig. 3. Data shown are mean ± SEM of *n* = 4, where each replicate was performed on a separate passage of cells. Significant differences are indicated by *, *p*<0.05; **, *p*<0.01; ***, *p*<0.001 and ****, *p*<0.0001 (versus DMSO control); or #, *p*<0.05; ##, *p*<0.01; ###, *p*<0.001 and ####, *p*<0.0001; analysed by one-way ANOVA with Tukey's post-test [Abbreviations: C1  =  class I inhibitor; LD  =  limit of detection].

LY294002 potently inhibited CXCL8 and CCL5 production in response to infection with RV-1B or RV-16 (Fig. 3). Class I PI3Ks have multiple isoforms. The pan class I inhibitor, PI-103, showed a similar pattern of inhibition of cytokine production to LY294002. However, when inhibitors targeting single isoforms were employed, we only observed significant inhibition of RV-1B-induced CCL5 production with β or δ inhibitors. We also studied these inhibitors in combination. These data (Fig. 4) showed the least inhibition of HRV-induced responses by α and γ combinations, but suggested roles for the other PI3K isoforms in responses to HRV. The more general class I inhibitor, PI-103, behaved more potently than combinations of individual class I inhibitors, giving it a profile more akin to that of LY294002 or 3-MA. Experiments in Figs. 3 and 4 were performed simultaneously, but results were divided over separate figures for clarity, resulting in duplication of the DMSO control bars and PI-103-alone bars in both figures.

**Figure 5:**
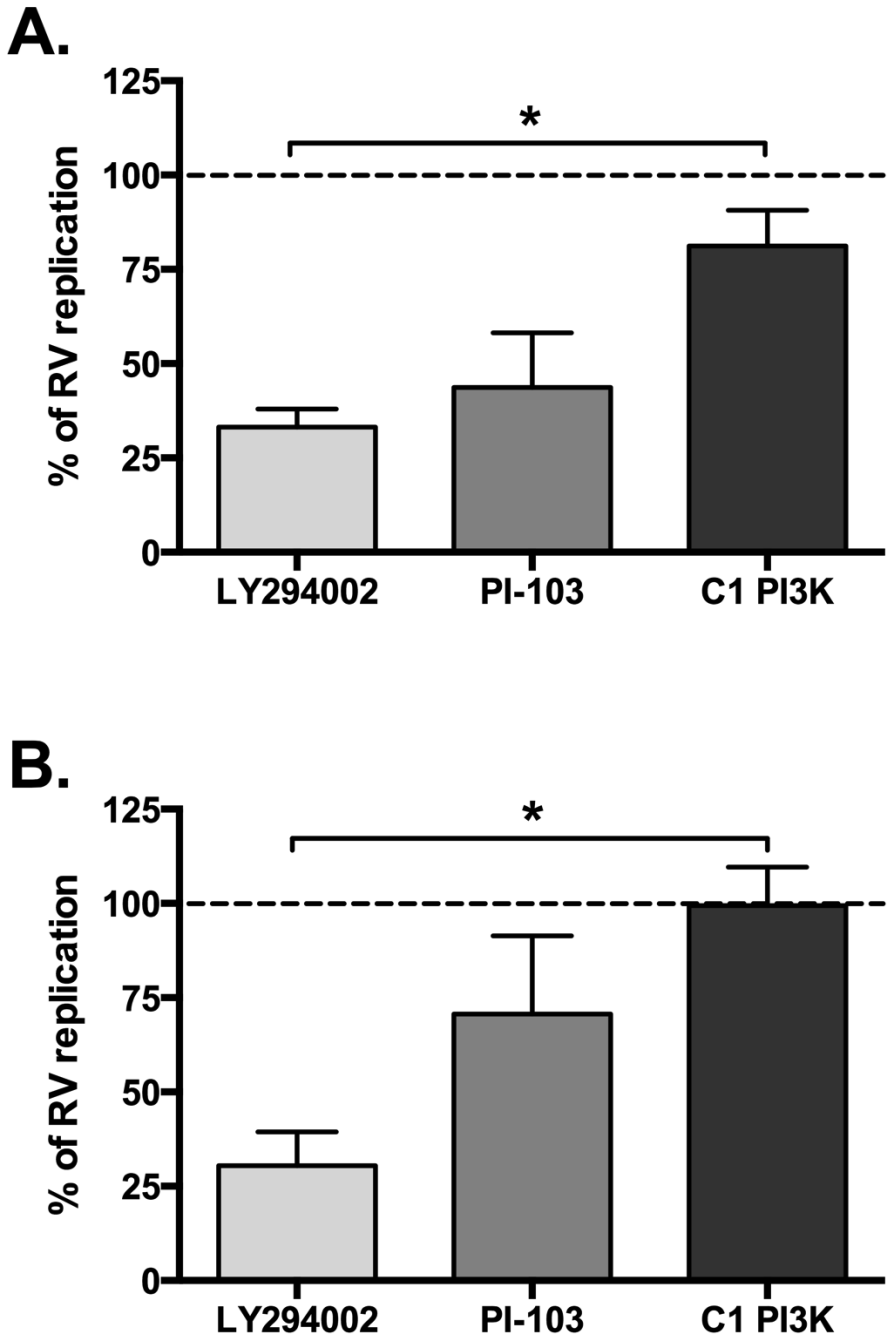
PI3K inhibitors LY294002 and PI-103 reduce viral replication. BEAS-2B cells were infected with RV-1B (A) or RV-16 (B) at 1×10^7^ TCID_50_/ml for 1 h (following which supernatants were replaced with media). Cells were then immediately treated with DMSO control, or with LY294002 (10 µM), PI-103 (0.5 µM) or a combination of four isoform-selective class I (C1) PI3K inhibitors [PIK-75 (α) (0.1 µM), TGX-221 (β) (0.1 µM), IC87114 (δ) (5 µM) and AS605240 (γ) (0.1 µM)]; and cultured for 24 h. RNA was extracted and reverse transcribed for qPCR analysis of HRV RNA expression, with data presented as the percentage of viral replication (normalised to DMSO control). Data shown are mean ± SEM of *n* = 4, where each replicate was performed on a separate passage of cells. Significant differences are indicated by *, *p*<0.05, analysed by one-way ANOVA with Tukey's post-test.

**Figure 6:**
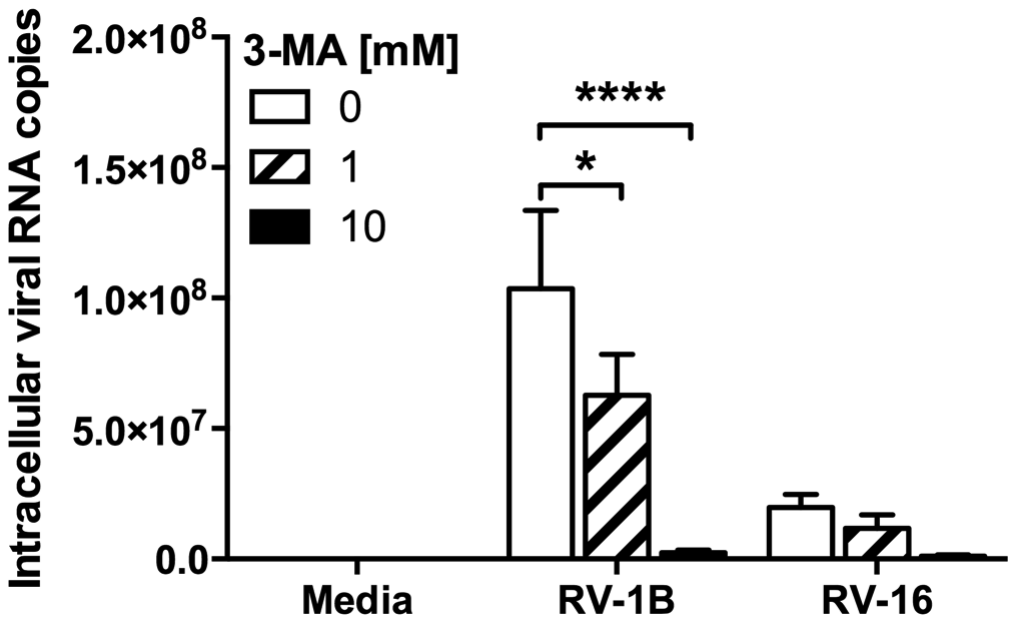
High concentrations of 3-MA inhibit viral replication. BEAS-2B cells were infected with RV-1B or RV-16 at 1×10^7^ TCID_50_/ml for 1 h (following which supernatants were replaced with media), and were then immediately treated with 3-MA at the indicated concentrations and cultured for 24 h. RNA was extracted and reverse transcribed for qPCR analysis of HRV RNA expression, with data presented as the total intracellular viral RNA copies per well. Data shown are mean ± SEM of *n* = 4 (A) or *n* = 3 (B), where each replicate was performed on a separate passage of cells. Significant differences are indicated by *, *p*<0.05 and ****, *p*<0.0001, analysed by two-way ANOVA with Bonferroni's post-test.

**Figure 7:**
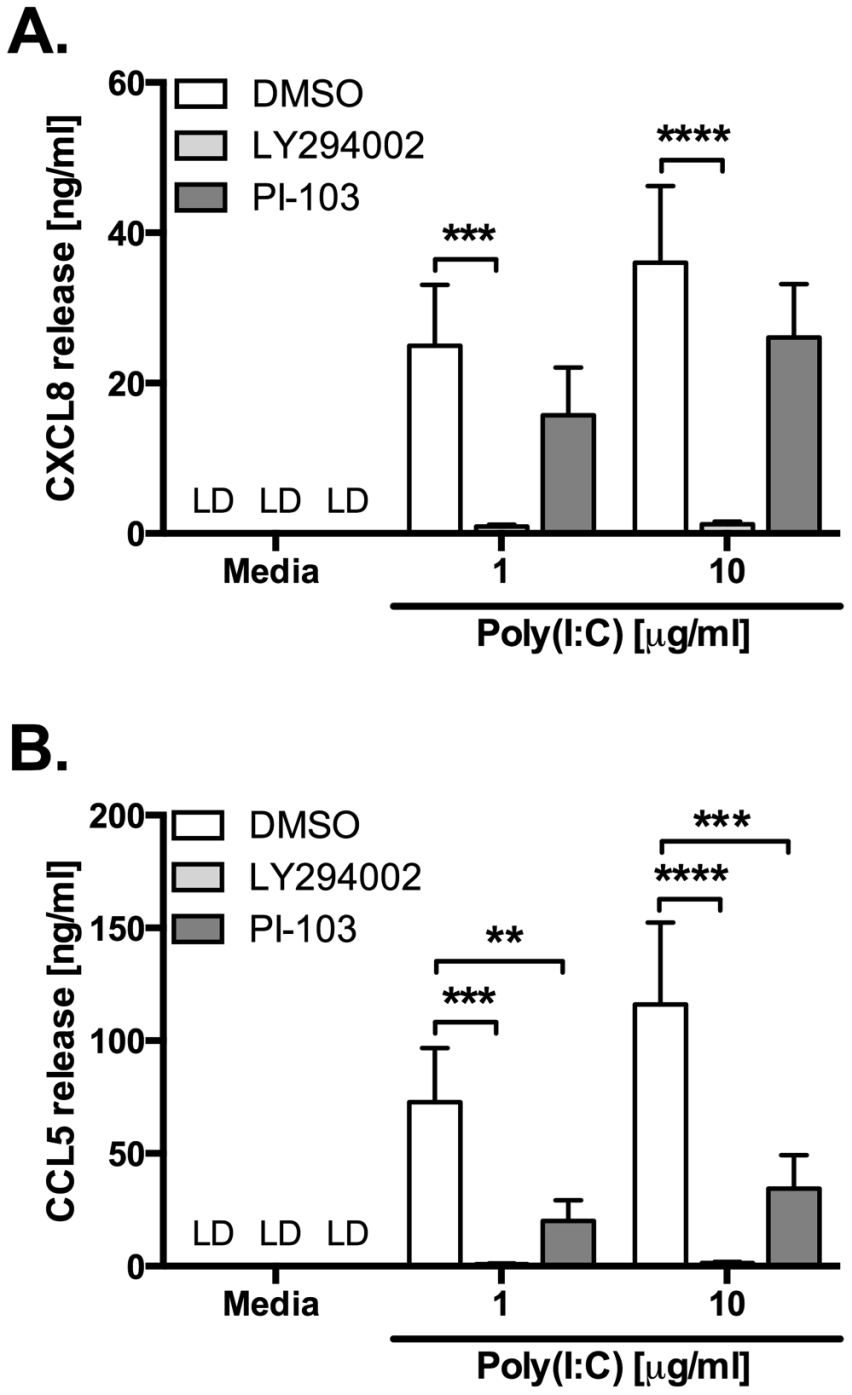
LY294002 and PI-103 inhibit proinflammatory cytokine production induced by poly(I:C). BEAS-2B cells were stimulated with poly(I:C) at the indicated concentrations and treated with DMSO control, or LY294002 (10 µM) or PI-103 (0.5 µM). After 24 h, cell-free supernatants were collected, and release of CXCL8 (A) and CCL5 (B) were measured by ELISA. Data shown are mean ± SEM of *n* = 4, where each replicate was performed on a separate passage of cells. Significant differences are indicated by **, *p*<0.01; ***, *p*<0.001 and ****, *p*<0.0001, analysed by two-way ANOVA with Bonferroni's post-test. LD  =  limit of detection.

PI3K inhibition has also been associated with modulation of viral replication. We observed that, although class I PI3K inhibition resulted in reduced cytokine production in response to HRV infection, a cocktail of class I selective inhibitors did not reduce viral replication (Fig. 5). However, PI-103 and LY294002 both markedly reduced viral replication, to a level which was statistically significant for LY294002 (Fig. 5). Similarly to PI-103 and LY294002, 3-MA also exhibited an ability to inhibit viral replication (Fig. 6). To try to determine the roles of PI3K in TLR3 signalling, without being confounded by a separate impairment of viral replication that could also reduce cytokine production, we examined the effects of PI-103 and LY294002 on poly(I:C)-induced cytokine production. Fig. 7 shows that both inhibitors reduced poly(I:C)-induced CXCL8 and CCL5 production, with LY294002 achieving complete inhibition of both cytokines, whilst PI-103 more potently inhibited CCL5 production, as compared to CXCL8, just as we observed in Fig. 1 using 3-MA.

**Figure 8:**
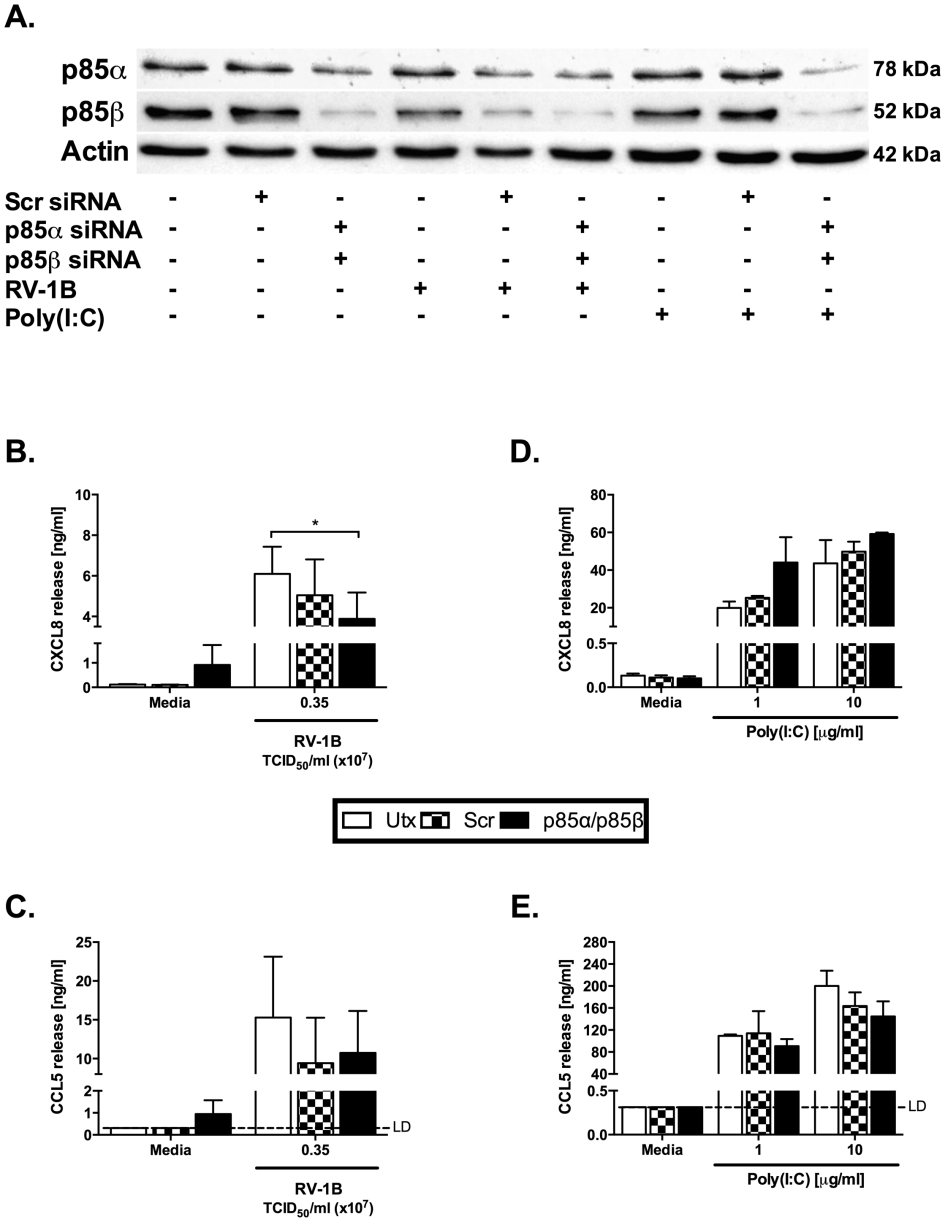
Dual knockdown of p85α and p85β modestly effects HRV-induced CXCL8 release. BEAS-2B cells were either left untransfected (Utx), transfected with a non-targeting scrambled control siRNA (Scr) or siRNA targeting p85α and p85β at 100 nM. After 48 h transfection, cells were infected with RV-1B at the indicated TCID_50_/ml for 1 h (following which supernatants were replaced with media) (B, C), or stimulated with poly(I:C) at the indicated concentrations (D, E). Cells were then cultured for 24 h. (A) Whole-cell lysates were analysed by western blot using Abs specific to p85α, p85β or actin. Data are representative of 3 (for HRV) and 2 (Poly(I:C)) independent experiments, each replicate was performed on a separate passage of cells. CXCL8 (B, D) and CCL5 (C, E) release was measured by ELISA. Significant differences (for HRV) are indicated by *, *p*<0.05 analysed by two-way ANOVA with Bonferroni's post-test. LD  =  limit of detection.

We attempted to validate the data obtained using small molecule inhibitors by other techniques. Initially we attempted dual siRNA knockdown for class I PI3K β and δ. These knockdowns could not be effectively validated at the protein level, as the commercially available δ antibodies we tested did not give clean enough blots to verify and quantify knockdown of these targets by siRNA. We therefore attempted knockdown of the key PI3K signalling protein, Akt. These knockdowns were associated with marked cell toxicity, and we therefore were unable to use these experiments to explore the roles of PI3K further. Finally, we attempted dual knockdown of the PI3K regulatory subunit proteins, p85α and p85β [22]. Fig. 8 shows that p85β levels were more easily reduced by siRNA than p85α, though neither protein could be reduced to undetectable levels. Knockdown of both adapters had modest effects on HRV-induced induced cytokine production (Fig. 8) and no effect on viral replication measured by qPCR (data not shown). Significant effects of dual knockdown, but not of relevant siRNA controls, were seen for virally-induced CXCL8 production only.

**Figure 9:**
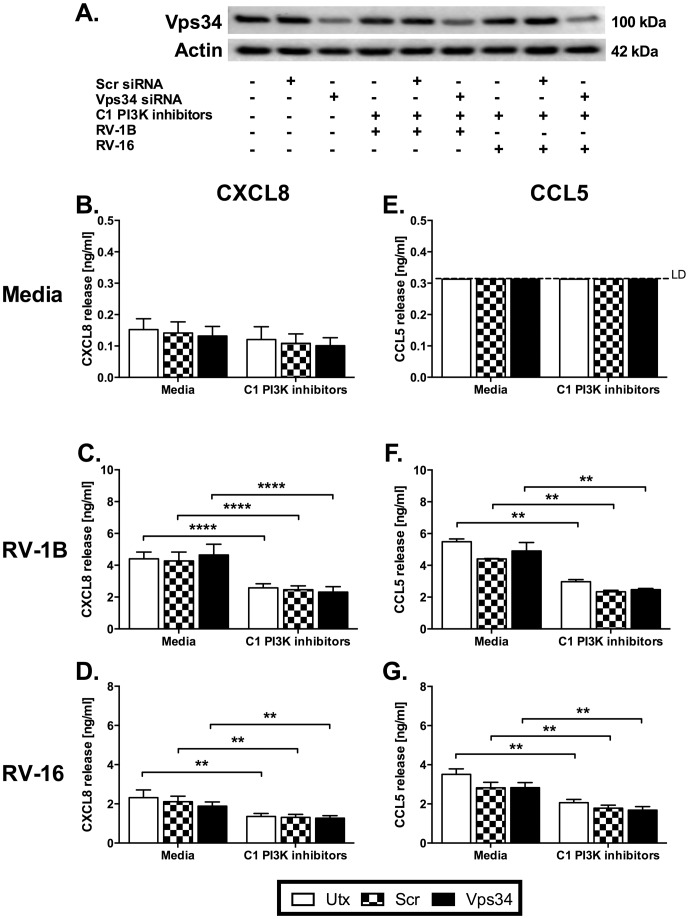
Knockdown of the sole class III PI3K, Vps34 does not inhibit HRV-induced cytokine release, and does not increase the inhibition of cytokine production achieved by a combination of isoform-selective class I PI3K inhibitors. BEAS-2B cells were either left untransfected (Utx), or transfected with a non-targeting scrambled control siRNA (Scr) or siRNA targeting Vps34 at 100 nM. After 48 h transfection, cells were infected with RV-1B (C, F) or RV-16 (D, G) at 1×10^7^ TCID_50_/ml for 1 h (following which supernatants were replaced with media). Cells were then either left untreated or treated with a combination of four isoform-selective class I (C1) PI3K inhibitors [PIK-75 (α) (0.1 µM), TGX-221 (β) (0.1 µM), IC87114 (δ) (5 µM) and AS605240 (γ) (0.1 µM)]; and cultured for 24 h. (A) Whole-cell lysates were analysed by western blot using Abs specific to Vps34 or actin. Image shown is a representative blot of levels of Vps34 knockdown. Cell-free supernatants were collected, and CXCL8 (B, C, D) and CCL5 (E, F, G) release was measured by ELISA. Data shown are mean ± SEM of *n* = 3, where each replicate was performed on a separate passage of cells. Significant differences are indicated by **, *p*<0.01 and ****, analysed by two-way ANOVA with Bonferroni's post-test. LD  =  limit of detection.

Since 3-MA is thought to target class III PI3K but may also target other PI3Ks, we sought to determine if combined targeting of class I and class III PI3K would recapitulate the phenotype observed with 3-MA by simultaneous use of the class I inhibitors with knockdown of the class III PI3K enzyme, Vps34. Knockdown of Vps34 had no effect on cytokine production induced by RV-1B or RV-16 infection, and did not increase the inhibition of cytokine production achieved by a combination of class I PI3K inhibitors (Fig. 9), indicating no involvement of class III PI3K in the responses to RV, and further indicating that 3-MA was likely to be exerting actions through modulation of other PI3K signalling.

### A possible role for mTOR in TLR3 signalling

**Figure 10:**
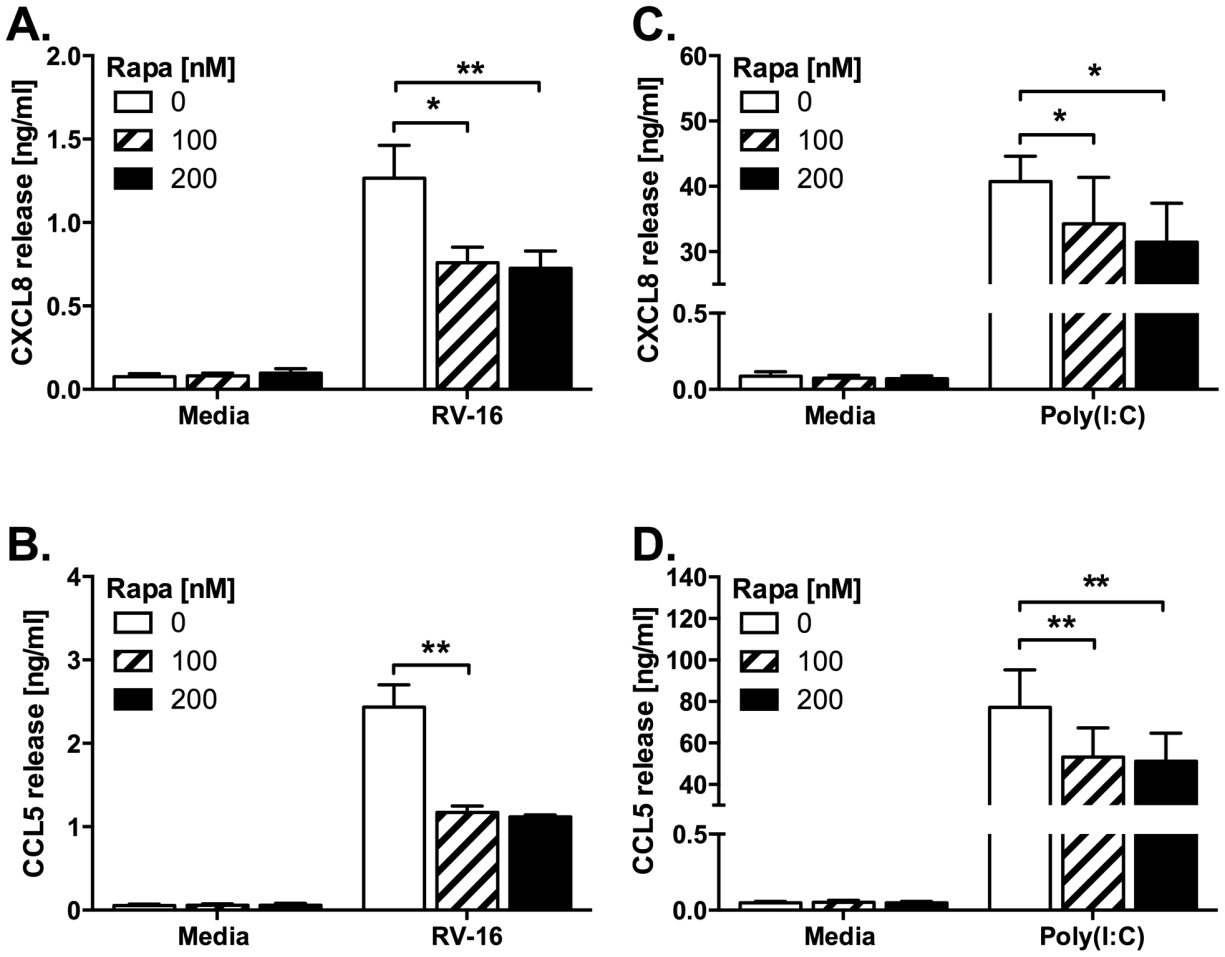
Rapamycin reduces proinflammatory cytokine production induced by HRV infection. BEAS-2B cells were infected with RV-16 at 1×10^7^ TCID_50_/ml for 1 h (following which supernatants were replaced with media) (A, B), or stimulated with poly(I:C) at 10 µg/ml (C, D). Cells were then immediately treated with rapamycin at the indicated concentrations and cultured for 24 h. Cell-free supernatants were collected, and release of CXCL8 (A, C) and CCL5 (B, D) were measured by ELISA. Data shown are mean ± SEM of *n* = 3, where each replicate was performed on a separate passage of cells. Significant differences are indicated by *, *p*<0.05 and **, *p*<0.01, analysed by two-way ANOVA with Bonferroni's post-test.

PI3K and mTOR pathways are closely intertwined and mTOR is subject to direct and indirect inhibition by compounds used in this study [23]. We considered that a component of the effects of the inhibitors we used could be mediated by mTOR inhibition. Results in Fig. 10 show that rapamycin inhibited CCL5 production, and to a lesser extent CXCL8 production, induced by RV16 and poly(I:C), suggesting possible roles for mTOR in the inflammatory responses of epithelial cells to rhinovirus infection.

**Figure 11:**
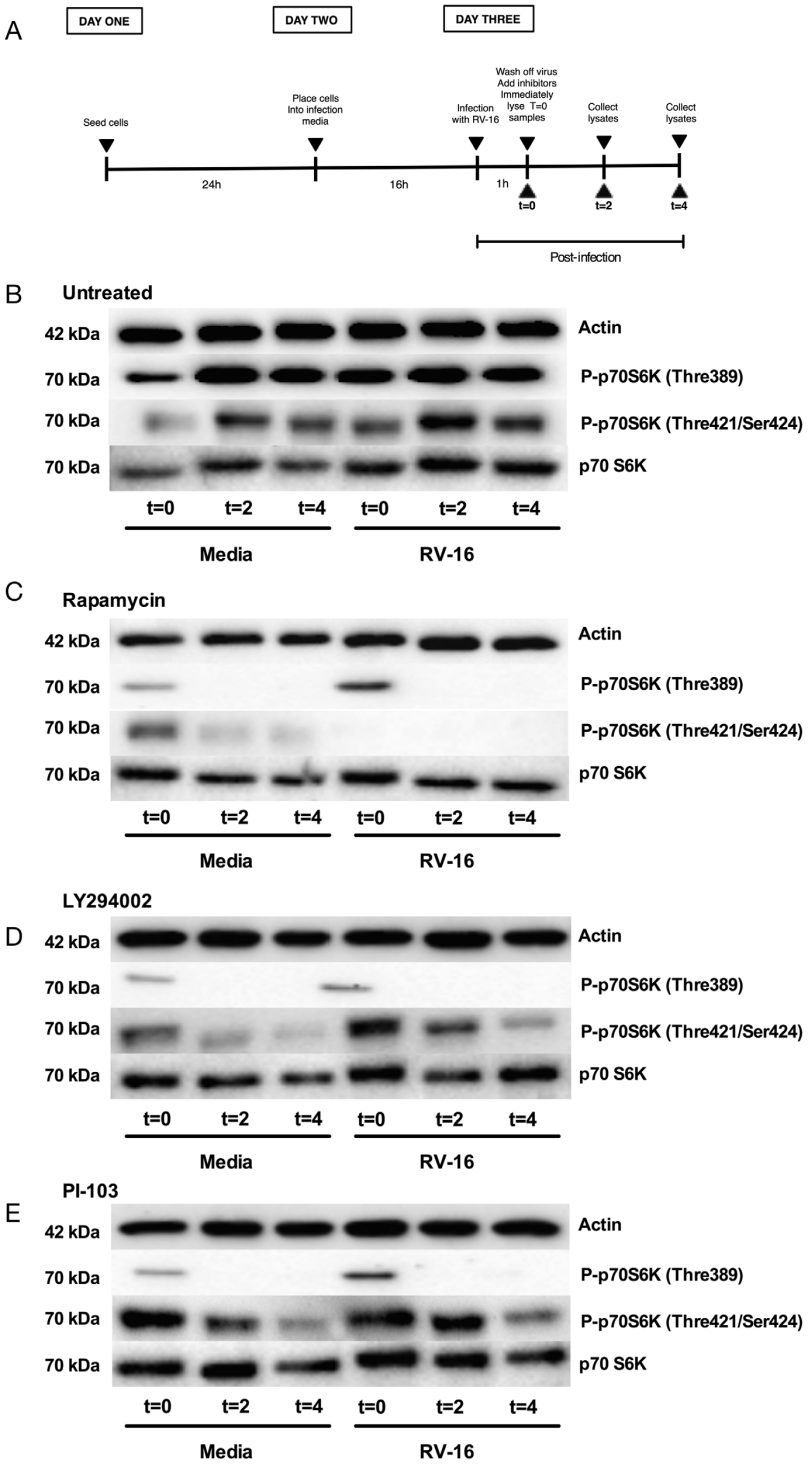
Rapamycin, LY294002 and PI-103 inhibit p70S6K phosphorylation. BEAS-2B cells were infected with RV-16 at 0.35×10^7^ TCID_50_/ml for 1 h, following which virus was removed and fresh media added. Panel A outlines the experimental design. Cells were then treated with Media (B), Rapamycin (100 nM) (C), LY294002 (10 µM) (D) or PI-103 (0.5 µM) (E), with whole-cell lysates collected immediately and designated t = 0, or collected at t = 2 and t = 4 (experimental plan shown in A). Lysates were analysed by western blot using Abs specific to phospho-p70S6K (Thre389), phospho-p70S6K (Thre421/Ser424), total p70S6K or actin. Blots representative of 3 independent experiments for each treatment are shown.

We therefore investigated whether exemplars of the PI3K pathway inhibitors we studied did indeed modulate mTOR activation. We examined the induction of the TOR complex target p70S6K whose activation is regulated by multi-site phosphorylation (inc Thr389 and Thr421/Ser424) [24]. We found that the changes of media during the experimental protocol induced changes of p70S6K phosphorylation. Rapamycin, LY294002, and the pan class I inhibitor PI-103 all inhibited p70S6K phosphorylation (Fig. 11).

### Knockdown of autophagy regulators has little effect on responses to HRV

Having observed that 3-MA inhibited cytokine generation induced by multiple agonists, we wished to determine the extent to which it might be mediating its effects by modulating autophagy.

**Figure 12:**
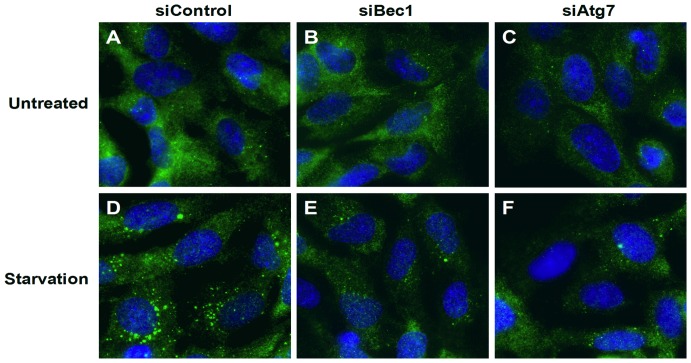
Knockdown of Beclin-1 or Atg7 inhibits basal and nutrient-starvation-induced autophagy in BEAS-2B cells. BEAS-2B cells were seeded on glass cover slips and transfected with a non-targeting scrambled control siRNA (siControl) (A, D), or siRNA targeting Beclin-1 (siBec1) (B, E) or Atg7 (siAtg7) (C, F) at 100 nM. After 48 h transfection, cells were either left untreated (A-C), or nutrient-starved (immersed in HBSS buffer) for 2 h (D-F). Cells were then fixed and immunostained for endogenous LC3 (green) and nucleus (blue). Images were captured using fluorescence microscopy at ×60 objective lens magnification. The green LC3 puncta represent autophagosomes. Representative images of 3 independent experiments are shown, where each replicate was performed on a separate passage of cells.

**Figure 13:**
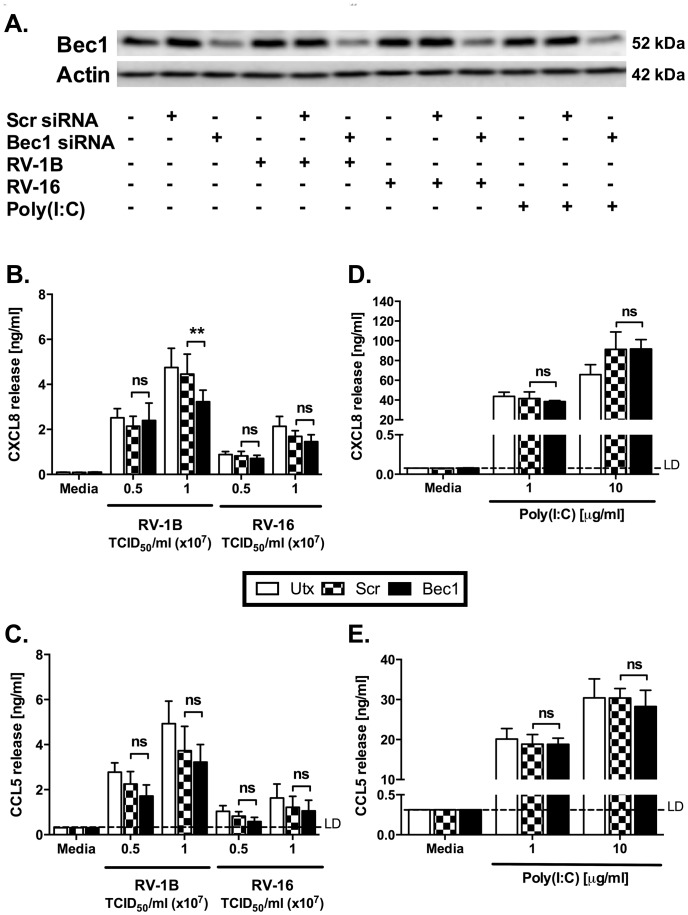
Knockdown of Beclin-1 has modest effect on HRV-induced cytokine release. BEAS-2B cells were either left untransfected (Utx), or transfected with a non-targeting scrambled control siRNA (Scr) or siRNA targeting Beclin-1 (Bec1) at 100 nM. After 48 h transfection, cells were infected with RV-1B or RV-16 at the indicated TCID_50_/ml for 1 h (following which supernatants were replaced with media) (B, C), or stimulated with poly(I:C) at the indicated concentrations (D, E); and cells were then cultured for 24 h. (A) Whole-cell lysates were analysed by western blot using Abs specific to Bec1 or actin. A blot representative of 3 independent experiments is shown. Data shown for RV-1B and RV-16 are using a TCID_50_/ml of 0.5×10^7^, and for poly(I:C) is using a concentration of 1 µg/ml. Cell-free supernatants were also collected, and CXCL8 (B, D) and CCL5 (C, E) release was measured by ELISA. Data shown are mean ± SEM of *n* = 3, where each replicate was performed on a separate passage of cells. The only significant difference on chosen statistical testing is indicated by **, *p*<0.01, and ns indicates no significant difference, analysed by two-way ANOVA with Bonferroni's post-test. LD  =  limit of detection.

The canonical pathway of macroautophagy requires the class III PI3K, Vps34, governed by its binding partner Beclin-1 and is dependent upon Atg7 for LC3 processing [25], [26]. To study the roles of autophagy in more detail, we transfected epithelial cells with siRNAs to autophagy proteins, in accordance with methods established in our lab [16]. We transfected cells 48 hours before viral infection, at which time point preliminary data had confirmed that effective knockdown of autophagy proteins had occurred (data not shown). Cells were then infected with HRV, and cytokine production and ongoing knockdown of autophagy proteins assessed 24 hours after viral infection. We observed that marked knockdown of Beclin-1 or Atg7 resulted in reduction in the endogenous LC3 processing induced by nutrient starvation (Fig. 12), thus we could be sure that we were able to inhibit canonical autophagy with these knockdowns. Additionally we found that Beclin-1 knockdown remained evident, albeit incomplete, at 24 h after viral infection (Fig. 13, i.e. 72 h after siRNA transfection).

**Figure 14:**
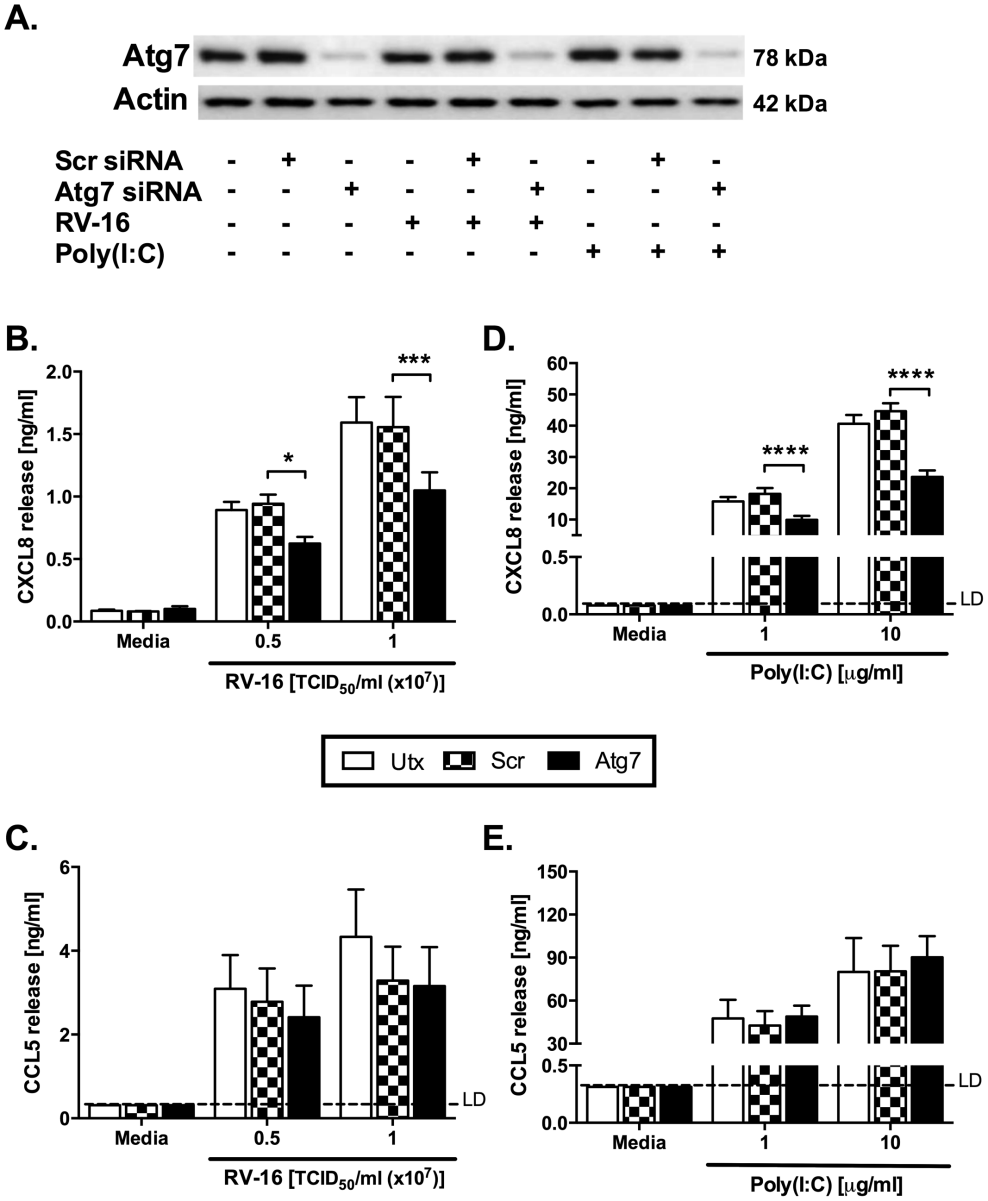
Knockdown of Atg7 modestly inhibits proinflammatory cytokine production induced by HRV infection. BEAS-2B cells were either left untransfected (Utx), or transfected with a non-targeting scrambled control siRNA (Scr) or siRNA targeting Atg7 at 100 nM. After 48 h transfection, cells were infected with RV-16 at the indicated TCID_50_/ml for 1 h (following which supernatants were replaced with media) (B, C), or stimulated with poly(I:C) at the indicated concentrations (D, E); and cells were then cultured for 24 h. (A) Whole-cell lysates were analysed by western blot using Abs specific to Atg7 or actin. A blot representative of 4 independent experiments is shown. Data shown for RV-16 are using a TCID_50_/ml of 0.5×10^7^, and for poly(I:C) is using a concentration of 1 µg/ml. Cell-free supernatants were also collected, and CXCL8 (B, D) and CCL5 (C, E) release was measured by ELISA. Data shown are mean ± SEM of *n* = 4, where each replicate was performed on a separate passage of cells. Significant differences are indicated by *, *p*<0.05; ***, *p*<0.001 and ****, *p*<0.0001, analysed by two-way ANOVA with Bonferroni's post-test. LD  =  limit of detection.

Despite marked knockdown of Beclin-1 protein expression, the data in Fig. 13 show only modest reduction of CXCL8 production in response to RV-1B only, with no effects on production of CXCL8 or CCL5 induced by RV-16 or poly(I:C). Knockdown of Atg7 was associated with moderate reductions in CXCL8 production induced by RV16 infection or poly(I:C) (Fig. 14). Again, induction of CCL5 by these stimuli was unaffected by Atg7 knockdown.

**Figure 15:**
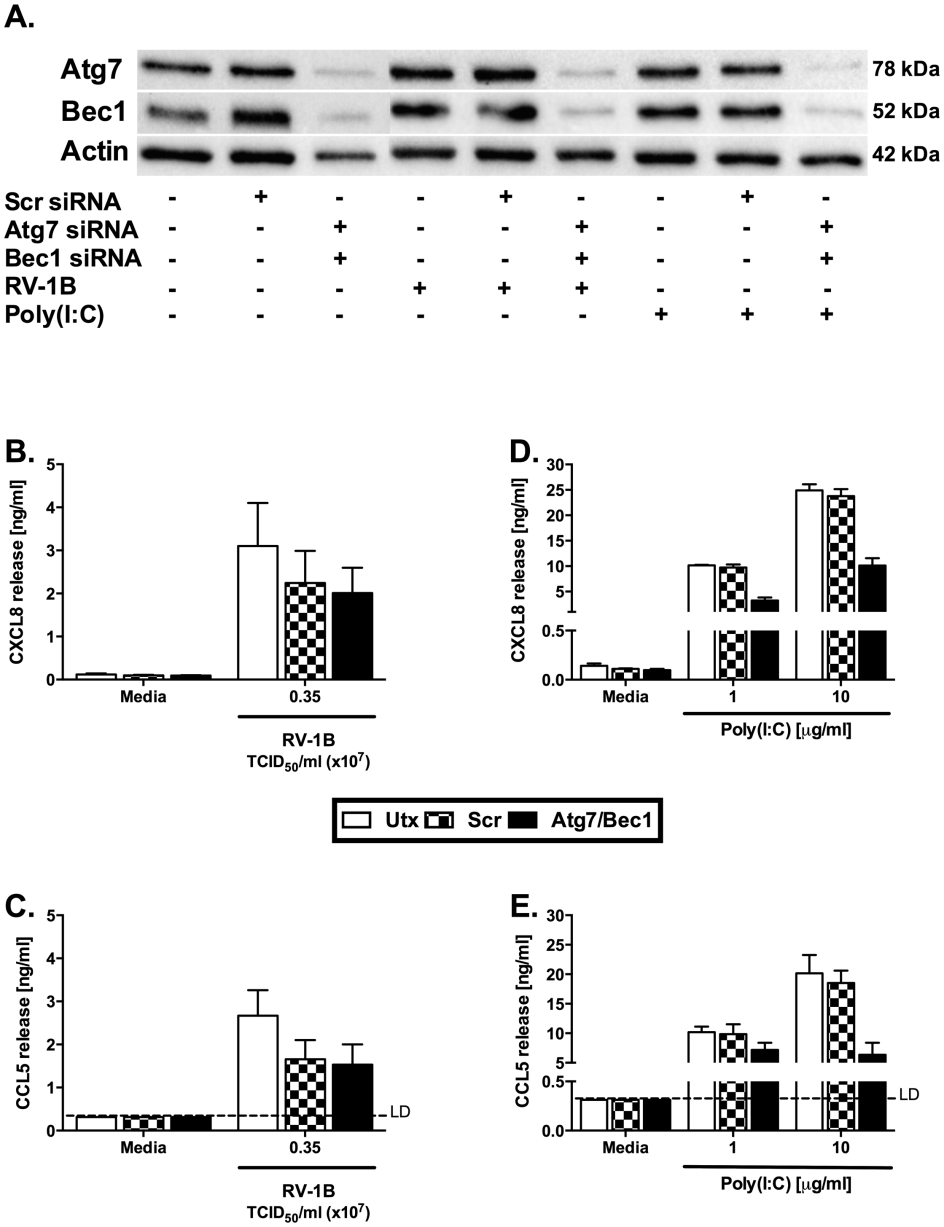
Dual knockdown of Atg7 and Beclin-1 modestly effects Poly(I:C)-induced cytokine release but not HRV-induced cytokine release. BEAS-2B cells were either left untransfected (Utx), transfected with a non-targeting scrambled control siRNA (Scr) or siRNA targeting Atg7 and Beclin-1 (Bec1) at 100 nM. After 48 h transfection, cells were infected with RV-1B at the indicated TCID_50_/ml for 1 h (following which supernatants were replaced with media) (B, C), or stimulated with poly(I:C) at the indicated concentrations (D, E). Cells were then cultured for 24 h. (A) Whole-cell lysates were analysed by western blot using Abs specific to Atg7, Bec1 or actin. A blot representative of 3 (for HRV) and 2 (Poly(I:C)) independent experiments is shown. CXCL8 (B, D) and CCL5 (C, E) release was measured by ELISA. Data shown are mean ± SEM of *n* = 3 for HRV and *n* = 2 for Poly(I:C). No significant differences for HRV were detected. LD  =  limit of detection.

To increase our targeting of the autophagy pathways, we performed experiments with dual siRNA knockdown of Beclin-1 and Atg7. Fig. 15 shows no additional effects on HRV-induced cytokine production when both siRNAs were combined.

siRNA targeting of autophagy proteins had modest effects on CXCL8 production, whilst 3-MA inhibited CCL5/IFN generation more effectively than CXCL8 production. Non-canonical pathways of autophagy can bypass Beclin-1 (reviewed in [27]), but LC3 remains critical to autophagy [26]. To be sure RV and poly(I:C) did not induce a non-canonical autophagy susceptible to 3-MA but not Beclin-1/Atg7 knockdown, we targeted LC3A&B by siRNA. Knockdown of LC3 did not affect production of CXCL8 and CCL5 induced by HRV infection (data not shown).

**Figure 16:**
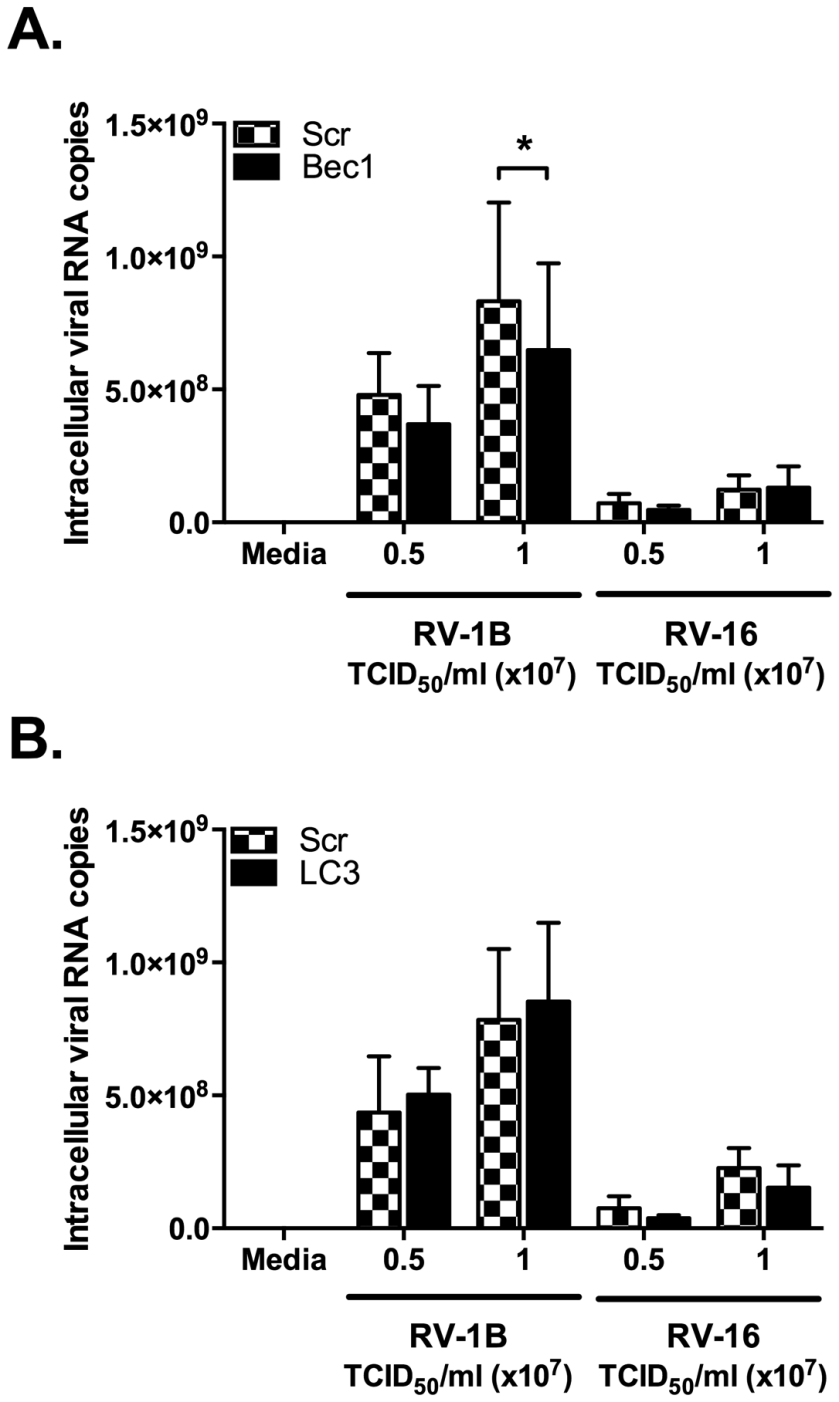
Knockdown of autophagy pathway proteins has minimal consequences for viral replication. BEAS-2B cells were transfected with a non-targeting scrambled control siRNA (Scr), or siRNA targeting Bec1 (A) or LC3 (B) at 100 nM. After 48 h transfection, cells were infected with RV-1B or RV-16 at the indicated TCID_50_/ml for 1 h (following which supernatants were replaced with media), and cultured for 24 h. RNA was extracted and reverse transcribed for qPCR analysis of HRV RNA expression, with data presented as the total intracellular viral RNA copies per well. Data shown are mean ± SEM of *n* = 3, where each replicate was performed on a separate passage of cells. The only significant difference on chosen statistical testing is indicated by *, *p*<0.05, analysed by two-way ANOVA with Bonferroni's post-test.

In some studies autophagy has also been implicated in the control of HRV replication [9], [11]. To investigate if changes were occurring in viral replication in the absence of regulation of cytokine production, we determined if knockdown of Beclin-1 or LC3 modulated replication of RV-1B or RV-16, measured by qPCR, a technique directly measuring production of viral RNA and correlating with the production of viral particles [17], [28]. Little effect on viral replication was observed with knockdown of either gene, with Beclin-1 knockdown achieving statistically significant, but modest, knockdown of viral replication at one TCID_50_ value of RV-1B only (Fig. 16).

## Discussion

The HRVs are an important family of human pathogens, and the dominant viral pathogen accounting for exacerbations of asthma and COPD. Here we show that PI3Ks have roles in the inflammatory responses to HRV infection and IFN signalling, and that these actions appear likely to be mostly independent of the important pathway of autophagy.

The PI3K family has many members, comprising multiple class I cytoplasmic enzymes, membrane-associated class II enzymes, and the single mammalian class III enzyme, Vps34, which regulates endosomal trafficking and autophagy [29]. We initially studied whether responses to HRV were modulated by 3-MA, a class III PI3K inhibitor widely used to probe autophagy. 3-MA inhibited HRV-induced production of CXCL8, the IFN-stimulated gene (ISG) CCL5, and IFNβ. However, the top concentration of 3-MA we used also inhibited responses to IL-1β, which suggested to us that the compound was likely to be having effects not mediated through autophagy.

Using broad inhibitors, a role for the PI3K family has been shown to have roles in responses to respiratory viruses including rhinovirus and influenza [3]–[6]. We therefore considered that 3-MA might be inhibiting other targets than class III PI3K, and compared the actions of 3-MA with those of a general PI3K inhibitor, LY294002 [19], [30], and a panel of class I isoform inhibitors. We observed that single inhibition of β or δ class I isoforms had modest effects on HRV-induced CCL5 production, and we also noted that CCL5 production was more sensitive to inhibition by 3-MA than CXCL8 production. These data suggest to us that at least some of 3-MA's actions may be mediated through inhibition of class I PI-3 kinases, in keeping with other recent published data [13]. When we used isoform-selective inhibitors in combination, α and γ combinations had the least efficacy, reinforcing roles for β and δ isoforms in responses to HRV. The only combination of selective inhibitors modulating CXCL8 production was that which targeted all of α, β, δ and γ isoforms, suggesting functional redundancy amongst these isoforms, as has recently also been noted for their roles in the control of neutrophil apoptosis [21].

The general class I inhibitor, PI-103, behaved differently from the isoform-selective class I inhibitors, with a more 3-MA and LY294002-like action. We noted that LY294002, PI-103, and 3-MA can all inhibit class I and class III PI3Ks [13], [20], [30], [31], suggesting that responses to HRV could be dependent upon both PI3K classes. However, we used PI-103 at 0.5 µM, and its IC_50_ for class III PI3K is 2.3–4 µM [20], [31], suggesting that PI-103 was not likely to be acting on class III PI3K in our experiments.

A significant limitation of these studies is that, although they used specific and validated inhibitors, small molecule inhibitors often exhibit effects on other targets than the intended molecule. We attempted to validate our findings using siRNA approaches. Unfortunately we were unable to demonstrate effective knockdown of PI3K class Iδ. Other approaches to target PI3K signalling were associated with cellular toxicity (siRNA to Akt). We attempted dual knockdown of the α and β subunits of the p85 regulatory PI3K subunit, and only saw minimal effects on HRV-induced cytokine production. However, we were unable to achieve complete knockdown of these adapters, and it is possible that effective doses of small molecule inhibitors achieved a greater proportional inhibition of PI3K than partial knockdowns of protein expression.

LY294002 has very recently been shown to inhibit TLR3-mediated activation of IFN independently of PI3Ks [32]. We noted that LY294002 and PI-103 both inhibit mTOR at concentrations used in our work [20], [23] and 3-MA can inhibit mTOR via inhibition of PI3K function [13]. The activation of mTOR is required for IFN generation in response to activation of TLR3 in keratinocytes [33] and TLR9 in DCs [34]. These data raise the possibility that activation of mTOR, independently of its roles in autophagy, may have important roles in responses to HRV infection. We found that the PI3K inhibitors LY294002 and PI-103 were able to inhibit phosphorylation of the mTOR target p70S6K. Rapamycin also inhibited cytokine induction in response to HRV infection and poly(I:C). These data indicate that some of the activities of PI3K inhibitors on the inflammatory response to HRV infection may be mediated by direct or indirect, PI3K-dependent inhibition of mTOR activation.

Although engagement of antiviral signaling activates PI3K [3], [5], we also note that inhibition of PI3K also modulates viral endocytosis [35]. Our data showed that broad spectrum, non-class I PI3K-selective inhibitors also reduced amounts of replicating virus, potentially compatible with effects on endocytosis and infection. However, they also inhibited responses to the synthetic agonist poly(I:C), suggesting direct regulation of TLR3 signalling pathways, although we cannot exclude the possibility that effects of PI3K inhibition may also impair delivery of poly(I:C) to endosomes.

The process of the PI3K-dependent pathway autophagy has clear roles in the responses to various viral infections [36]. In dendritic cells, autophagy can deliver viral RNAs to the intracellular TLRs [8]. Pathogens can also hijack autophagy, as illustrated by poliovirus which exploits autophagy for its replication [11]. The roles for autophagy in responses to HRVs are contradictory [9], [11]. We therefore determined whether autophagy was implicated in regulating the responses to HRV infection, and whether regulation of autophagy explained any actions of PI3K inhibitors. To achieve this, we targeted several members of the autophagy pathway, alone or in combination, by siRNA.

We transfected cells with siRNAs 48 hours before viral infection, at which point pilot data indicated we had good knockdown of the target proteins. We subsequently evaluated the consequences of knockdown of target proteins on responses to HRVs at 24 hours after viral infection (72 hours after transfection of siRNA). We did not achieve total knockdown of our targets, but we achieved levels of knockdown that were similar to those we previously found sufficient to explore the roles of Pellino-1 and RIP-1 [16], and which reduced autophagy induced by the classical stimulus of nutrient starvation. Knockdown of Beclin-1 or Atg7, alone or in combination, had variable modest effects on induction of CXCL8 by HRV. Knockdown of LC3 had no effects on responses to either virus (data not shown). Viral replication was only minimally modified by Beclin-1 knockdown. Although it is possible that we missed uncovering a role for autophagy through not achieving complete knockdown of Beclin-1 or Atg7, overall these data indicate that autophagy plays only modest roles in responses to commonly studied major and minor HRVs. Furthermore, inhibition of autophagy has most effect on the signalling pathway from TLR3 to CXCL8 production, an effect that could result from modulation of IL-1β [37], [38], whose roles in HRV-induced inflammation we have described previously [17], [39].

In conclusion, our data showed that whilst direct inhibition of autophagy had some consequences for HRV-induced CXCL8 production, its contributions to HRV-induced inflammation in these systems were modest, although we note that remaining low levels of autophagy proteins may permit some responses. Furthermore, we show for the first time roles for, and redundancy amongst, class I PI3Ks in the control of HRV infection, and we suggest a role of mTOR in the regulation of responses to HRV.
